# The Role of Functional Health Literacy in terms of Harmful Alcohol Use in Adults with Probable Posttraumatic Stress Disorder and Alcohol Use Disorder

**DOI:** 10.21203/rs.3.rs-4144996/v1

**Published:** 2024-03-26

**Authors:** Dylan A. Gould, Rebecca E. Lubin, Shelby J. McGrew, Tanya Smit, Anka A. Vujanovic, Michael W. Otto, Michael J. Zvolensky

**Affiliations:** Boston University; Boston University; Texas A&M University; University of Houston; Texas A&M University; Boston University; University of Houston

**Keywords:** Functional health literacy, post-traumatic stress disorder, alcohol use disorder, hazardous alcohol use

## Abstract

**Purpose:**

The current study examined functional health literacy (FHL) in regard to hazardous drinking among a sample with probable posttraumatic stress disorder (PTSD) and alcohol use disorder (AUD).

**Methods:**

Participants were 565 adults with probable PTSD and hazardous alcohol use (52.2% female, 68.8% Non-Hispanic White, average age = 39.2 years ± 10.9 years).

**Results:**

FHL literacy maintained statistically significant role in terms of hazardous drinking (*p* < .001) even in the context of posttraumatic stress.

**Conclusion:**

FHL may be important to better understand hazardous drinking among persons with comorbid PTSD and AUD.

## 1. Introduction

The co-occurrence of posttraumatic stress disorder (PTSD) and alcohol use disorder (AUD) is a prevalent and pernicious comorbidity (1). PTSD and AUD have a lifetime prevalence of 8% and 29%, respectively (2,3). The prevalence of comorbid hazardous alcohol use among individuals with PTSD is estimated to range from 9.8–61.3% and the prevalence of comorbid PTSD in those with hazardous alcohol use ranges from 2.0–63.0% (4). Comorbid PTSD and AUD are associated with more severe symptoms when compared to the presence of one condition (5–7). Other work indicates that comorbid PTSD and AUD are related to poorer mental health (5–7), more frequent suicide attempts (5,8), increased unemployment, reduced higher education, and higher relationship instability (9). Although adverse life events are frequently linked to PTSD (10) and AUD (11), little effort has focused on the role social determinants of health (SDoH) related to hazardous drinking among individuals with these comorbid conditions.

Health literacy is a SDoH construct that reflects the capacity of an individual to obtain, interpret, and understand basic health information and services in ways that are health-enhancing (12). Lower health literacy is associated with poor health status, higher mortality, decreased preventative service usage, deficient disease self-management, poor health behaviors, and higher healthcare costs (13,14). Functional health literacy (FHL) is a subtype of health literacy that pertains to the ability to apply relevant health information, such as health risks, healthcare utilization, and coping mechanisms, to a health situation (15). FHL may play a role in hazardous drinking among persons with comorbid PTSD and AUD given that FHL has been associated with increased alcohol use in adolescent, adult, and elderly populations (16–18), although contradictory evidence does exist (19,20). To our knowledge, no work has explored FHL in terms of hazardous drinking among persons with comorbid PTSD and AUD.

The current study evaluated FHL in terms of hazardous drinking among adults with probable PTSD and AUD. Because past work has found higher FHL is associated with health-promoting behaviors (21) and lower FHL is related to greater alcohol use (16–18), it was hypothesized that FHL will demonstrate a statistically significant effect for hazardous drinking in the context of PTSD symptom severity.

## 2. Methods

### 2.1 Protocol

The current study is a secondary analysis of data from a project studying the transdiagnostic risk and maintenance factors of PTSD and hazardous alcohol use among people with probable PTSD and AUD. The participants for this project consist of a nationally representative sample of adults recruited through Qualtrics Panels, an online data collection platform. Participants first completed eligibility screening questionnaires and then provided voluntary informed consent to participate in the study. Eligibility criteria for the study were as follows: being between the ages of 18 and 65, having access to a computer or mobile device, endorsing symptoms consistent with probable PTSD based on a score of 3 or above on the *Primary Care PTSD Screen* (PC-PTSD; 22), and having probable AUD based a score of at least 3 for females or 4 for males on the three-item *Alcohol Use Disorders Identification Test* (AUDIT-C; 23). The exclusion criteria for the study were inability to provide voluntary informed consent, inability to complete online surveys, and lack of English proficiency. Participants were compensated for completing the baseline assessment through cash-based incentives, reward miles, or reward points. This study was approved by the University of Houston Institutional Review Board.

### 2.2 Participants

Participants included 565 who screened positive for probable PTSD and AUD. The biological sex composition was 52.2% female. The racial and ethnic composition was 68.8% Non-Hispanic White, 15.2% Black or African American, 8.8% Hispanic White, 3.0% Asian, 1.4% Native American/Alaskan Native, 0.5% Native Hawaiian or Pacific Islander, and 2.1% Other. The mean age of the sample was 39.2 years (*SD* = 10.9).

### 2.3 Measures

*2.3.1 Demographics* were measured using a self-report questionnaire that included age, biological sex, gender identity, ethnicity, race, education, occupation, living situation, and income.

*2.3.2* The *All Aspects of Health Literacy Scale* (AAHLS; 24) is a 14-item, self-report questionnaire used to assess health literacy. In the current study, the FHL subscale score was used, which is derived by calculating the mean of the three health literacy items of the subscale. Cronbach’s α for this subscale, using items 1 and 3 in accordance with Chin & McCarthy (24), was .803.

*2.3.3* The *Alcohol Use Disorders Identification Test* (AUDIT; 25) is a 10-item self-report questionnaire used to assess alcohol consumption, drinking behaviors, alcohol-related problems, hazardous alcohol use, and harmful alcohol use. In the current study, the AUDIT total score was employed to assess hazardous alcohol use. This scale demonstrated good internal consistency (Cronbach α = .894).

*2.3.4* The *PTSD Checklist for DSM-5* (PCL-5; 26) is a 20-item self-report questionnaire used to assess past month PTSD symptom severity. Participants identified their worst traumatic event using the *Life Events Checklist for DSM-5* (LEC-5; 27) and indicated their level of distress for that event for each symptom using a 5-point Likert-type scale ranging from 0 (*not at all*) to 4 (*extremely*). The items were summed to create a total score. This item demonstrated excellent internal consistency (Cronbach α = .964).

### 2.4 Data Analytic Plan

First, predictor variables of interest (PCL and FHL) were mean centered. Second, correlation coefficients were calculated to examine the associations between the study variables. Third, linear regressions were conducted regressing AUDIT total score onto PCL total symptoms and FHL total score. Finally, the interaction term between FHL and the PCL was added in the third step of the regression models. Age, biological sex, and race were included as covariates in the models. Participants who had missing data on variables of interest were excluded from the analyses.

## 3. Results

### 3.2 Preliminary Analyses

Pearson correlations were calculated to examine relationships between age, FHL, PCL, and AUDIT scores. Point-biserial correlations were calculated to examine relationships between continuous variables with race and sex. Means (*M*), standard deviations (*SD*) for continuous variables and intercorrelations are presented in Table 1.

### 3.3 Regression Analyses

Both PCL (β = .355, t = 9.263, *p* < .001) and FHL (β = − .144, t = −3.721, *p* < .001) scores were statistically significantly associated with AUDIT total scores, together predicting 17.6% of the variance in AUDIT scores. FHL accounted for between 1.6–5.9% of variance in the model depending on whether PCL is included. Regarding covariates, male sex (β = − .266, t = −7.248, *p* < .001) also emerged as a significant predictor of the AUDIT total score. The interaction term was not found to be a statistically significant predictor of the AUDIT score (β = .043, t = 1.169, *p* = .243).

## 4. Discussion

Results indicated that there was a main effect for FHL for hazardous drinking even in the context of a statistically significant effect for PTSD symptom severity. There was no evidence of an interaction between FHL and PTSD symptom severity for hazardous drinking. These data suggest that FHL may be an underrecognized SDoH in the context of PTSD that may be useful for better understanding hazardous drinking among persons with comorbid PTSD and AUD. The current findings extend past work that has reported a negative association between FHL and higher alcohol use (16–18). The mechanism pertaining to the FHL-hazardous drinking effect is not clear, but lower health-information seeking (28) may impair acquisition of adaptive coping abilities, thereby increasing the risk for hazardous alcohol use.

The present findings have clinical and public health implications. FHL screenings may be considered for implementation by clinicians who see individuals presenting PTSD and hazardous alcohol use. Further, there may be merit to offering greater attention to improving alcohol health literacy as part of prevention (19, 29), consistent with earlier recommendations (30).

Several study limitations should be noted. First, data were collected using self-report measures and future research could benefit by employing a multimethod assessment protocol. Second, the sample was recruited from the general population, but was not treatment-seeking; consequently, future research could be extended to samples seeking treatment for hazardous drinking. Third, the sample was predominantly White, limiting generalizability to more racially and ethnically diverse populations. Third, motivation to drink was not assessed in the current study, and therefore, the motivational bases of alcohol use could not be determined. Future research could explore drinking motives as explanatory factors in terms of FHL and hazardous drinking.

## 5. Conclusion

FHL was a statistically significant predictor of hazardous alcohol use among individuals with probable PTSD and AUD. FHL, a prevalent SDoH construct, could represent an intervention target for mitigating hazardous drinking among persons with PTSD and comorbid AUD.

## Figures and Tables

**Table 1:** Descriptive Statistics and Correlations Among Study Variables

	*M*	*SD*	Age	Sex	Race	PCL	FHL	AUDIT-C
Age^1^	39.17	10.88	-					
Sex^1^	-	-	.03	-				
Race^1^	-	-	− .15***	.00	-			
PTSD Symptom Severity^2^	46.27	19.99	− .13**	− .02	.02	-		
FHL^3^	2.47	0.48	.10*	.15***	− .07	− .30***	-	
Hazardous Alcohol Use^4^	18.72	9.61	− .10*	− .29***	.07	.41***	− .29***	-

* *p* < .05;

** *p* < .01;

*** *p* < .001; 1 = Demographics questionnaire; PCL = The PTSD Checklist for DSM-5; 3 = All Aspects of Health Literacy Scale; 4 = Alcohol Use Disorders Identification Test
